# Genital Giant Molluscum Contagiosum in an Immunocompetent Male Patient: A Case Report and Literature Review

**DOI:** 10.7759/cureus.88410

**Published:** 2025-07-21

**Authors:** Yoshihiro Ono, Yoshiyuki Miyazawa, Seiji Arai, Yoshitaka Sekine

**Affiliations:** 1 Department of Urology and Venereology, Maebashi Primary Clinic, Maebashi, JPN; 2 Department of Urology, Gunma University School of Medicine, Maebashi, JPN

**Keywords:** genital, giant molluscum contagiosum, immunocompetent, pox virus, sexually transmitted diseses

## Abstract

Molluscum contagiosum (MC) is an epidermal infectious disease caused by DNA molluscipoxvirus. Genital MC is well known as a sexually transmitted disease occurring in adulthood. A 27-year-old male presented with a two-month history of a growing pinkie-sized genital mass. At the right base of the penis, a dome-shaped 1.5 cm nodule was present with two typical MCs nearby. A giant genital MC was diagnosed, which was gently and bluntly plucked off with forceps without pain or bleeding. The PubMed and Google Scholar databases were searched using the keyword "giant molluscum contagiosum," and profiles of 104 cases were confirmed. Genital MCs are rare in immunocompetent patients.

## Introduction

Molluscum contagiosum (MC) is an epidermal infectious disease caused by a molluscipoxvirus that usually presents as small, asymptomatic, skin-colored umbilicated papules 1-3 mm in diameter. The condition most commonly occurs in disseminated forms [1-3]. Although it mainly affects children, sexually active adults and immunocompromised patients, such as those with human immunodeficiency virus (HIV) infection, are also at risk [2,3]. Cases are defined as “giant” MC when the lesions exceed 5 mm in children or 1 cm in adults [1-3]. We present an adult case of a solitary giant MC on the genital area of an immunocompetent man. We have also compiled a mini-literature review.

## Case presentation

**Figure 1:**
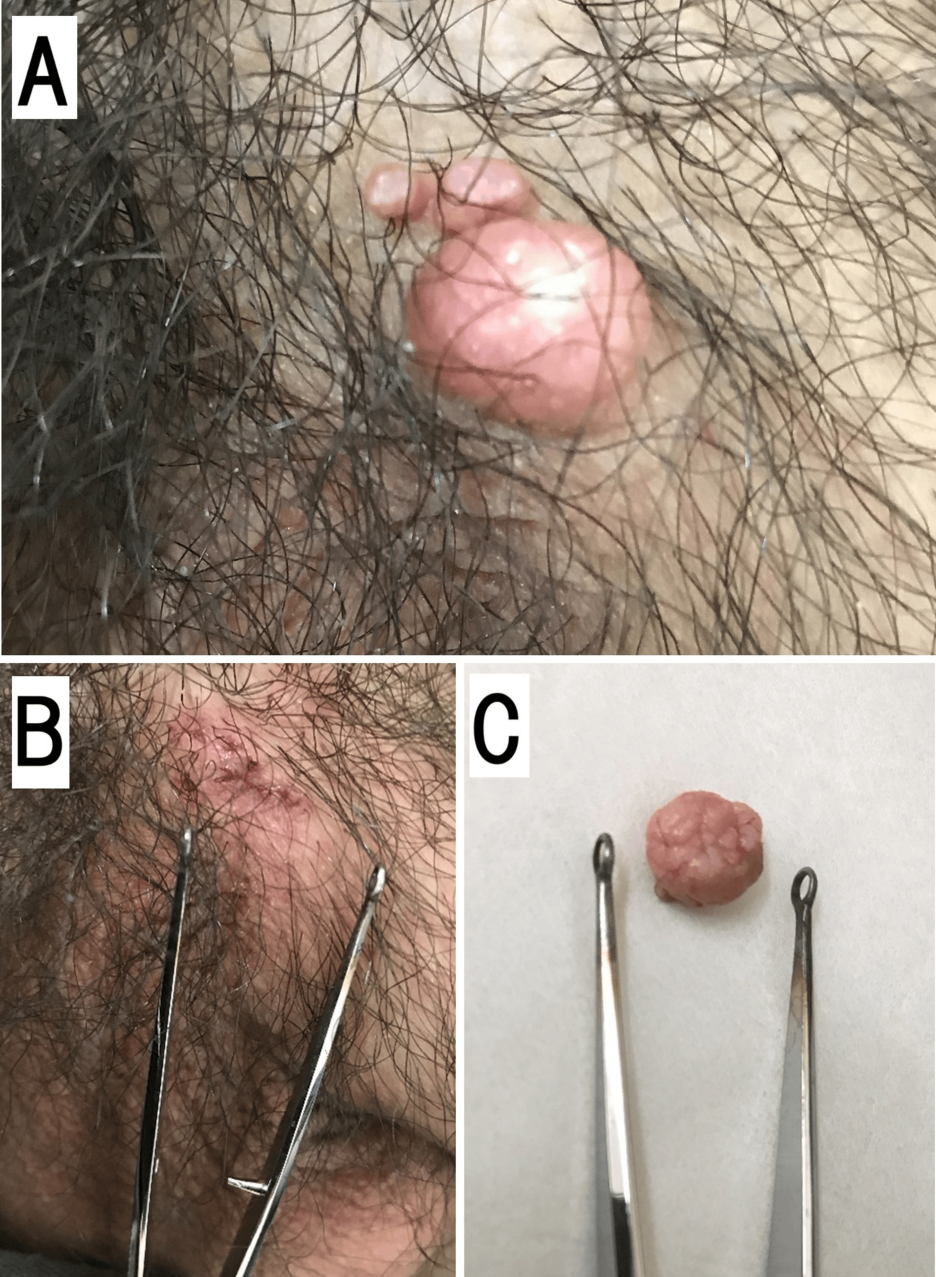
Macroscopic appearance of the giant genital MC. Macroscopic appearance of giant genital MC. (A) Dome-shaped nodule 1.5 cm in diameter and two typical MCs with an umbilicated center at the right side of the base of the penis. (B) Scar immediately after blunt plucking of a giant MC without bleeding. (C) Giant MC immediately after removal. MC: Molluscum contagiosum

A 27-year-old man presented with a two-month history of an expanding pinkie-sized genital mass. A dome-shaped 1.5-cm nodule was present on the right side of the base of the penis. In addition, there were two skin-colored papules (3 mm and 4 mm) with umbilicated centers typical of MC nearby (Figure 1A). The two typical MCs were removed using curettage. Examination of the larger nodule revealed that the epidermis at the base of the dome was very soft, brittle, and fragile. However, it lacked an apparent umbilical center; therefore, giant MC was diagnosed. Differential diagnoses included epidermoid cysts, abscesses, granulomas, and lipomas. The lesion was gently and bluntly plucked off with forceps without bleeding or pain (Figures 1B, 1C).

The patient had been in a monogamous relationship for three years; however, his partner's history of MC was unknown. We advised the patient that his partner should also be checked for the disease; however, it is uncertain whether she did so. He had no previous history of unprotected sexual intercourse or sexually transmitted diseases, including MC. On physical examination, he was healthy with no abnormal findings, and HIV antigen-antibody test results were negative.

**Figure 2:**
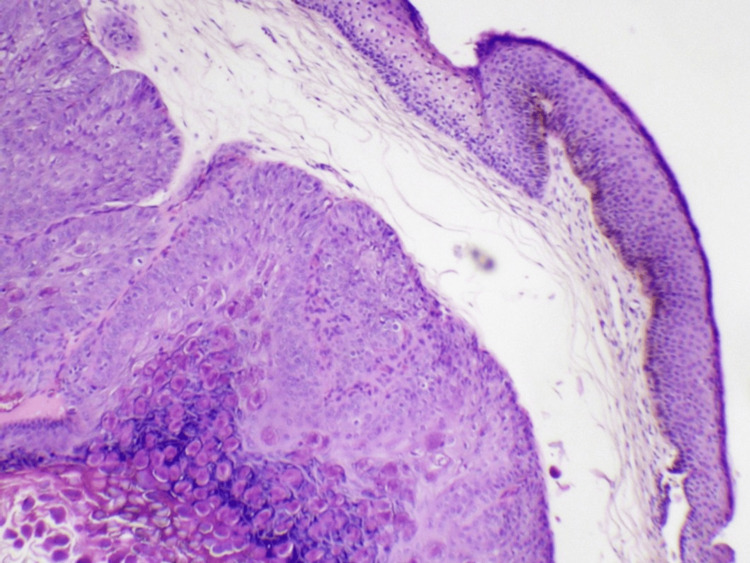
Histopathological findings (hematoxylin and eosin staining, magnification 200x). Histopathological findings (hematoxylin and eosin staining, magnification 200x). A suprabasilar epidermal lesion consisting of “molluscum bodies” and small eccentric nuclei are present with no significant inflammation.

Histopathological examination revealed a suprabasilar epidermal lesion consisting of large molluscum bodies with granular and eosinophilic cytoplasmic inclusions. No significant inflammation was observed (Figure 2).

The post-treatment course was uneventful, with no recurrence during the three-month follow-up observation period.

## Discussion

Genital MC is well-known as a sexually transmitted disease of adulthood [1,2]. Established therapies for genital MC include curettage, cautery, and cryotherapy [1]. The choice of treatment depends on factors such as cost, the number, size, and sites of lesions, patient immune status, and physician experience. In a study of patients with genital MC, Balkrishna et al. reported that 7 of 25 patients (28%) with HIV presented with the giant form, compared to only one of 25 cases (5%) without HIV [2]. While the giant form of genital MC is very rare, careful vigilance is required as it can indicate HIV infection or other immunocompromised conditions [4,5]. For example, one report described a refractory case of acute myeloid leukemia in which giant MCs had spread to the whole body. The patient required extensive medical treatment, including acitretin, radiofrequency ablation, and potassium hydroxide intralesional injections [6]. In another case, a patient had HIV along with giant MC and opportunistic complications, leading to a poor prognosis without the opportunity to receive appropriate treatment for HIV and giant MC [3]. Improving the immune function of patients with giant MC with appropriate antiviral drugs is important, especially in those with HIV [1,2]. Matsuda et al. emphasized that patients with multiple or giant MC cannot be considered immunocompetent until a detailed immunological evaluation is performed, even if they are HIV-negative and have no history of immunocompromising diseases [5]. In the current case, no immunological abnormalities, including HIV, were present.

We searched PubMed and Google Scholar using the keywords "giant molluscum contagiosum" and found 145 publications (as of April 1, 2025). We determined that 98 of the articles were suitable for identifying case profiles. Among those cases, 50 described patients who were immunocompetent, of which 14 were ≥18 years of age [7-19] (Table 1).

**Table 1:** A compilation of variables using published articles on case reports of giant MC in immunocompetent patients. Source: [7-19]

	Over all
Variables	n=14+1 (this case)
Sex	
Male	7 (50%)
Female	7 (50%)
(N/A)	1
Age in years (median)	17–68 (49)
Number of giant MCs	
single	9 (60%)
>1 (multiple)	6 (40%)
Max. diameter in cm (median)	1.0–4.0 (1.2)
Sites	
Face/Head/Neck	8 (53%)
Arm/Hand/Leg/Foot	5 (33%)
Trunk/Buttock	3 (20%)
Genital	2 (13%)
Number of sites	
One site	13 (87%)
Two sites	1 (7%)
Three sites	1 (7%)
All four sites	0
MC, molluscum contagiosum; N/A, not applicable.	

The gender ratio was equal. Forty percent were cases of multiple forms, and most occurred at a single anatomical site. Two of 15 (13%) cases (including our current case) of giant MC were on genital sites. Hassan et al. reported another case with giant and multiple MCs on three sites, including the genitals. The case was diagnosed after confirming that tests for HIV, viral hepatitis, and other autoimmune diseases were negative and immunoglobulin fraction and lymphocyte subset tests were normal [7]. Therefore, this mini-literature review supported rarity of giant MCs, particularly on the genitals, in immunocompetent individuals.

When giant MCs do appear in immunocompetent individuals, their prognosis is good with proper treatment [1,7]. However, Viswanath et al. reported a case of multiple disseminated giant MCs on the face of an immunocompetent adult which was extremely difficult to treat [8]. Therefore, although the condition is very rare, strong attentiveness is required, as multiple giant MCs at multiple anatomical sites may be refractory even in immunocompetent patients. In addition, cases of multiple genital giant MCs, genital giant MCs at multiple sites, genital giant MCs along with dissemination of ordinary MCs, or refractory and recurrent MCs are indicators of an immunocompromised condition.

## Conclusions

Here, we report a rare case of giant genital MC in a 27-year-old immunocompetent man. The clinical features of giant MC were analyzed through a literature review from which we highlighted and clarified valuable aspects of disease management. Genital giant MCs in immunocompetent patients have good prognosis with local treatment. However, vigilance is crucial in cases of multiple forms or giant MCs with associated lesions at other sites, as these can indicate an underlying immunocompromised state that may lead to refractory disease.
